# College and Hospital Notes

**Published:** 1902-05

**Authors:** 


### COLLEGE AND HOSPITAL NOTES.

At the Buffalo Hospital of the Sisters of Charity an examination was held April 23, 1902, for six internes, the following-named being the successful candidates: J. R. Sackrider, East Aurora; Albert Frye, Buffalo; Charles Haase, Elmira; J. J. Brown, Wellsville; E. Haley, Buffalo, and Thomas J. Walsh, Buffalo; Messrs. Brown, Haley and Walsh enter upon their duties in May, and the others November 1, 1902.

The German Hospital has recently put into service a handsome new ambulance of the most approved pattern. It has all the equipment essential and is of the most modern construction. Ventilation is provided in the sides of the vehicle. For the ease of the patients the cot is suspended from the roof and smooth running will be secured by the rubber tires of the wheels. At the sides of the cot are adjustable seats for the surgeons.

The consumption hospital, which was built to replace the one destroyed by fire two years ago, has been completed at the county farm and was thrown open to the inspection of the public for the first time March 28, 1902.

The building cost $49,900 and its equipment $5,000 more. It has accommodations for 54 patients, which can be increased to 70 if especially required. The plans were drawn by architect Metzger. The building is two stories in height and is constructed of limestone quarried on the county farm.

Dr. E. J. Gilray, who has served in that capacity for some years, continues as resident physician in charge.

The Medical Department of the University of Buffalo will hold its fifty-sixth annual commencement at the Teck Theatre at 11 o'clock, on the morning of May 2, 1902. The Rev. Dr. David J. Burrell, of New York, will deliver the address to the graduates. The Alumni Association will hold its twenty-seventh annual meeting on the same day in the University building. The usual ceremonies will take place, including class reunions, luncheons and the annual dinner. The latter will be given at the Hotel Iroquois at 6.30 p.m., ladies being invited.

The scientific program will be called at Alumni Hall in the afternoon and the president, Dr. F. H. Moyer, of Moscow, will take the chair at 2 o'clock and deliver his address. Then will follow a symposium on gunshot wounds, to be participated in by Major Louis A. LaGarde, M.D., U.S.A., of the Soldiers' Home, Washington, D.C.; Lt.-Col. G. Sterling Ryerson, M.D., C.A.M.S., South African Contingency, Toronto; Maj. William P. Kendall, M.D., U.S.A., Fort Porter, Buffalo; Roswell Park, William Warren Potter, William C. Phelps, Herbert M. Hill, Ph.D., William H. Mosher, Phar. D., of Buffalo, and William H. Bergtold, of Denver, Col. The papers will be illustrated by stereopticon views, and in addition there will be an exhibition of guns, ancient and modern, projectiles and explosives.

---

A SUMMER school will be held at Syracuse University for six weeks, viz.: July 1 to August 9, 1902. This school is designed to accommodate (1) teachers of elementary and secondary schools; (2) students who may desire to review college preparatory work, or (3) to prepare for state preliminary examinations; (4) students desiring to do college work during the summer. Credit will be given for work under (4).

Libraries and laboratories will be open and available. Tuition for three or more courses, $25; for less, $10 a course. Thoroughly competent men will give the instruction. Courses will be offered in Greek, Latin, German, English, history, pedagogy, mathematics, physics and chemistry, and in other subjects if arranged for in advance. For circular and information, address Prof. J. R. Street, Ph.D., Director, Syracuse University.

---

THE faculty of the medical department of the University of Buffalo offers two prizes, a first of $15.00, and a second of $10.00, for the best college song which shall be suitable for the use of the alumni and students of the University of Buffalo.

The conditions of the competition are as follows: (*a*) the contest is open only to the alumni and students of the four departments of the University of Buffalo—medicine, pharmacy, law and dentistry; (*b*) copies must be in the hands of the judges on or before September 15, 1902, signed by the contestant, naming department and class year; (*c*) the judges to award the prizes are to be appointed by the faculty of the medical department of the University of Buffalo. Send all communications to executive committee, Alumni Association, University of Buffalo, Buffalo, N. Y.

